# Differences in global gene expression in muscle tissue of Nellore cattle with divergent meat tenderness

**DOI:** 10.1186/s12864-017-4323-0

**Published:** 2017-12-04

**Authors:** Larissa Fernanda Simielli Fonseca, Daniele Fernanda Jovino Gimenez, Danielly Beraldo dos Santos Silva, Roger Barthelson, Fernando Baldi, Jesus Aparecido Ferro, Lucia Galvão Albuquerque

**Affiliations:** 1 Faculty of Agricultural and Veterinary Sciences, São Paulo State University, FCAV/UNESP, Jaboticabal, São Paulo, Brazil; 2 CyVerse, University of Arizona, Tucson, USA

**Keywords:** RNA-Seq, Transcriptome, Meat quality

## Abstract

**Background:**

Meat tenderness is the consumer’s most preferred sensory attribute. This trait is affected by a number of factors, including genotype, age, animal sex, and pre- and post-slaughter management. In view of the high percentage of Zebu genes in the Brazilian cattle population, mainly Nellore cattle, the improvement of meat tenderness is important since the increasing proportion of Zebu genes in the population reduces meat tenderness. However, the measurement of this trait is difficult once it can only be made after animal slaughtering. New technologies such as RNA-Seq have been used to increase our understanding of the genetic processes regulating quantitative traits phenotypes. The objective of this study was to identify differentially expressed genes related to meat tenderness, in Nellore cattle in order to elucidate the genetic factors associated with meat quality. Samples were collected 24 h postmortem and the meat was not aged.

**Results:**

We found 40 differentially expressed genes related to meat tenderness, 17 with known functions. Fourteen genes were up-regulated and 3 were down-regulated in the tender meat group. Genes related to ubiquitin metabolism, transport of molecules such as calcium and oxygen, acid-base balance, collagen production, actin, myosin, and fat were identified. The PCP4L1 (Purkinje cell protein 4 like 1) and BoLA-DQB (major histocompatibility complex, class II, DQ beta) genes were validated by qRT-PCR. The results showed relative expression values similar to those obtained by RNA-Seq, with the same direction of expression (i.e., the two techniques revealed higher expression of PCP4L1 in tender meat samples and of BoLA-DQB in tough meat samples).

**Conclusions:**

This study revealed the differential expression of genes and functions in Nellore cattle muscle tissue, which may contain potential biomarkers involved in meat tenderness.

**Electronic supplementary material:**

The online version of this article (10.1186/s12864-017-4323-0) contains supplementary material, which is available to authorized users.

## Background

Meat quality traits in Brazilian animal breeding programs have not been fully explored because of the late expression of these attributes and the complex evaluation that can only be made after slaughter. Furthermore, on the domestic market, producers are generally not paid for meat quality, a fact that diminishes interest in improving meat quality traits and has hindered their inclusion in traditional selection objectives. In contrast, on international markets, meat tenderness is one of the most valued traits [39], a fact that highlights the importance towards improving this trait since Brazil is one of the world’s largest beef exporters.

Meat tenderness is the preferred sensory attribute of consumers [7]. According to Scollan et al. [46], the European food industry has sought to improve this trait to gain market share over other types of food. In Brazil, about 80% of the cattle herd consists of Zebu animals or their crossbreeds [1]. In this respect, the improvement of meat tenderness becomes important since Ferguson et al. [17] have shown that the higher the proportion of Zebu genes in a population, mainly Nellore cattle, the less tender the meat. Meat tenderness can only be measured after slaughter making this trait more complex to select animals. Thus, alternative tools are useful to include meat tenderness in animal breeding programs [9].

Modern recently developed large-scale RNA sequencing technologies (RNA-Seq) have been useful in understanding the genetic and physiological processes that regulate the phenotype of quantitative traits in a certain situation [34]. RNA-Seq permits analysis of the transcriptional profiles of cells, tissues or organs in a certain situation and the discovery of known and unknown genes involved in a given cellular process [57]. This new technique can be used to identify novel potential molecular markers that permit more accurate and early genetic predictions [51], with a consequent reduction in the generation interval that would contribute to the improvement of difficult-to-measure traits such as meat tenderness.

RNA-Seq has been widely used in recent studies to investigate differentially expressed genes related to meat tenderness in different species. For example, genes related to the degradation of filamins, lipogenesis and collagen synthesis have been identified in a study on meat tenderness in broiler chickens [40]. Gonçalves [20] found genes related to metabolic pathways involved in apoptosis, calcium transport, proteolysis and ribosome synthesis in castrated Nellore cattle, classified as extreme for tenderness based on estimated breeding values for shear force measured after 14 days of aging. Bongiorni et al. [8], who studied gene expression in longissimus dorsi muscle of animals of two Italian beef breeds (Maremmana and Chianina) representing the extremes for meat tenderness, detected differentially expressed genes related to growth and sodium-potassium pumps, among others.

Despite the above-mentioned publications, studies investigating differentially expressed genes related to meat tenderness in cattle are rare. In this respect, the better understanding and identification of the transcripts and biological processes, associated with this complex and economically important trait, will permit to highlight genes that could contain potential biomarkers involved in meat tenderness.

The objective of this study was to identify genes differentially expressed in muscle tissue (longissimus dorsi) of Nellore cattle with divergent meat tenderness using RNA-Seq in order to obtain data that increase our understanding of the genetic and metabolic mechanisms underlying this trait.

## Results

### RNA sequencing, alignment, and assembly of the transcripts

The TopHat2 program identified a total of 942 million reads (2 × 100 bp) and the sequencing coverage was 63X (coverage for all transcripts of all samples). An average of almost 24 million reads were obtained per sample and 88.3% of the reads were mapped. For tender meat group, an average of 24,928,506 (89%) million reads were mapped, while for tough meat group, an average of 22,170,021 million reads were mapped (89%) (Additional file 1: Table S1).

We found transcript for 28,059 genes and 103,309 potential new isoforms.

To evaluate the quality of sequencing, the expression profiles of the Glucuronidase Beta (GUSB), erythrocyte hydroxymethylbilane synthase (HMBS), Hypoxanthine Phosphoribosyltransferase 1 (HPRT1), phosphoglycerate kinase 1 (PGK1) and TATA-Box Binding Protein (TBP) genes were analyzed, which exhibited a similar expression profile in the tender and tough meat groups (Additional file 2: Figure S1).

A box plot (Additional file 3: Figure S2) containing the transformed FPKM values (log_10_) for each group and the plot of principal component analysis (PCA) (Additional file 4: Figure S3) were constructed using the cummerRbund package. As can be seen in the box plot, the distribution of quartiles was consistent between groups, indicating high quality of the data. In addition, the medians were similar in the two groups and close to −1, indicating that the level of sequencing coverage permitted the identification of low-expressed genes [11, 51]. PCA showed the formation of different groups (tender and tough meat), indicating differences in the expression of genes between the tender and tought meat groups’.

### Analysis of differentially expressed genes

Analysis of differential expression in the tender and tough meat groups revealed 40 differentially expressed genes (*q*-value <0.05) (Table 1). Seventeen of these genes have a known function. The log_2_ signal (fold change) was used was used to partition the DE genes into up- and down-regulated groups. In this analysis, 35 genes were found to be up-regulated and 5 were down-regulated in relation to the tough meat group. Among the genes with known function, 14 were up-regulated and 3 were down-regulated.

**Table 1:** Differentially expressed genes detected in the samples divergent for meat tenderness

Gene	Locus	Tender	Tough	log2 (fold change)^a^	*p*-value	*q*-value
XLOC_002455	10:2,270,299–2,271,226	213.386	0.108797	429.375	0.0001	0.0078
XLOC_014455	21:61,587,088–61,588,096	194.026	0.110438	413.493	0.0001	0.0078
XLOC_002491	10:20,795,303–20,795,839	49.187	0.291183	407.828	0.0001	0.0078
IQCG	1:70,792,451–70,838,105	0.975199	0.0683124	383.548	0.0001	0.0078
CLEC4G	7:17,785,971–17,789,026	213.916	0.152397	381.114	0.0002	0.0251
EXOSC2	11:101,010,350–101,010,501	146.306	109.334	374.217	0.0001	0.0078
DMGDH	10:9,994,009–10,066,954	0.700546	0.0710559	330.145	0.0001	0.0078
XLOC_029184	GJ060137.1:2576–3597	194.018	0.224785	310.957	0.0001	0.0078
CLDN19	3:104,091,609–104,096,449	112.997	0.133369	308.279	0.0001	0.0078
XLOC_006476	14:27,940,730–27,941,920	173.549	0.233603	289.322	0.0001	0.0078
USP32	19:13,087,794–13,088,565	293.099	0.401415	286.822	0.0001	0.0078
CTNNB1	22:13,897,328–13,898,816	129.849	0.179967	285.103	0.0001	0.0078
AT2	X:1,024,840–1,027,901	112.057	0.163197	277.955	0.0001	0.0078
XLOC_022848	4:115,985,386–115,986,391	206.906	0.302608	277.346	0.0001	0.0078
XLOC_028948	9:32,650,968–32,653,108	0.903283	0.135688	273.489	0.0001	0.0078
CLEC12A	5:100,388,588–100,401,353	205.909	0.31943	268.844	0.0001	0.0078
XLOC_024373	5:47,857,813–47,858,488	368.063	0.591044	263.861	0.0001	0.0078
ZKSCAN2	25:23,091,496–23,104,117	0.689172	0.130967	239.566	0.0001	0.0078
XLOC_022865	4:119,488,532–119,490,812	0.857893	0.171544	232.222	0.0001	0.0078
XLOC_022836	4:114,608,102–114,609,417	166.248	0.334759	231.214	0.0001	0.0078
SYP	X:92,308,615–92,320,415	0.814972	0.169198	226.804	0.0001	0.0078
XLOC_029021	9:76,153,645–76,154,023	105.826	23.362	217.945	0.0001	0.0078
XLOC_002519	10:28,447,880–28,449,134	173.421	0.402111	210.862	0.0001	0.0136
XLOC_021066	3:20,773,659–20,775,848	100.205	0.252969	198.593	0.0001	0.0078
XLOC_001046	1:150,301,045–150,303,606	0.781722	0.211096	188.876	0.0001	0.0136
ASAH1	27:18,312,322–18,314,792	0.924241	0.265766	179.811	0.0001	0.0136
XLOC_018808	28:17,838,746–17,847,124	120.255	0.353753	176.528	0.0001	0.0136
XLOC_001185	1:91,928,092–91,930,927	0.775044	0.232207	173.887	0.0001	0.0078
TCF7L1	11:49,714,024–49,715,693	931.924	299.392	163.818	0.0001	0.0078
XLOC_022736	4:59,964,007–59,966,831	0.820647	0.270615	160.052	0.0002	0.0198
XLOC_001280	1:151,442,513–151,443,449	221.613	0.163023	37.649	0.0001	0.0078
ENSBTAG00000022947	21:21,965,065–21,966,315	156.027	0.257492	25.992	0.0001	0.0078
LOC511981	14:58,850,047–58,851,086	300.014	108.315	14.698	0.0001	0.0136
PCP4L1	3:8,249,980–8,276,724	179.755	0.413335	2.120	0.0001	0.0078
BoLA-DQB	23:25,855,145–25,863,052	122.213	682.692	−0.840093	0.0001	0.0078
HMOX1	5:73,980,699–73,987,841	115.512	197.874	−0.776539	0.0002	0.0251
PNP	6:87,555,287–87,556,157	163.788	543.206	−159.226	0.0001	0.0078
XLOC_004829	13:42,396,473–42,396,753	241.093	676.471	−183.349	0.0001	0.0078
XLOC_029070	9:102,893,791–102,894,054	21.503	101.088	−441.086	0.0001	0.0078

Symbol of the differentially expressed gene (Gene), location of the gene in the *Bos taurus* genome, FPKM values obtained for tender and tough meat, relative expression (log_2_ fold change), *p*-value, and *q*-value

^a^The fold-change estimates (relative expression) refer to the tender meat group

Combined functional annotation using all differentially expressed (up- and down-regulated) genes for meat tenderness was performed with the DAVID v6.7 database using *Bos taurus* as a reference. This analysis permitted the identification of seven functional groups (annotation clusters; Additional file 5: Table S2). These genes were classified according to their function: cell fraction (GO: 000267), cell junction (GO: 0030054), intrinsic component of membrane (GO: 0031224), regulation of cell communication (GO: 0010646), catalytic activity (GO: 0003824), organelles (GO: 0043226), and binding (GO: 0005488), among others.

**Fig. 1:**
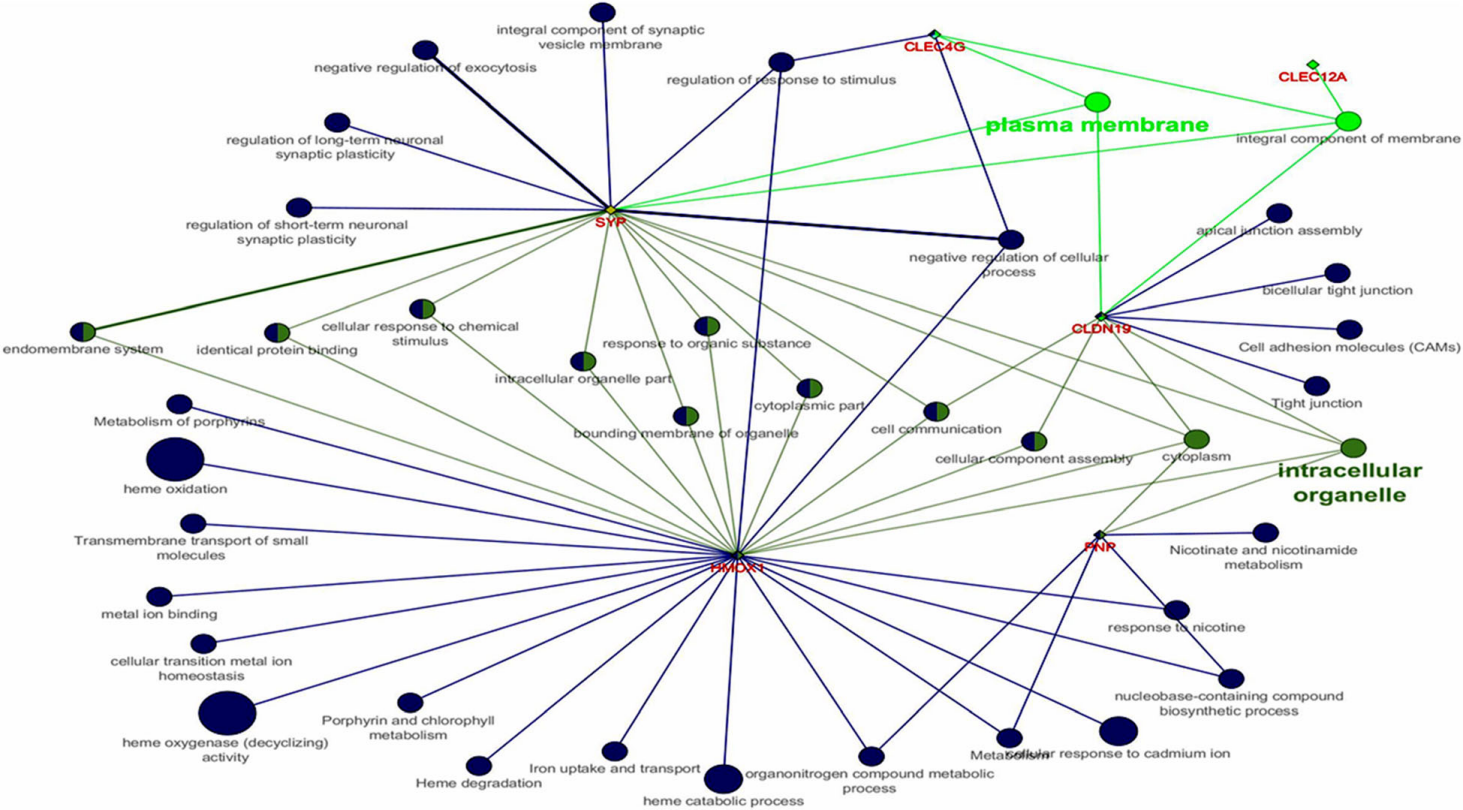
Enrichment analysis of the HMOX1, CLDN19, CLEC4G, CLEC12A, PNP and SYP genes using the ClueGO plug-in of the Cytoscape program. Note the interrelationships between these genes, which are related to the transport of molecules

Using the ClueGO plug-in, the differentially expressed transcripts HMOX1, AT2, CLDN19, CLEC4G, CLEC12A, PNP and SYP were found to be inter-related through biological processes (cell communication, regulation of response to stimuli), molecular function (binding proteins), or cell components (integral membrane component) (Fig. 1). The gene HMOX1 was expressed more in tough meat group, while the other five genes were expressed more in tender meat group. The proteins encoded by these transcripts are involved in the transport of molecules such as sodium, potassium, calcium, and oxygen [15, 29, 36, 43].

**Fig. 2:**
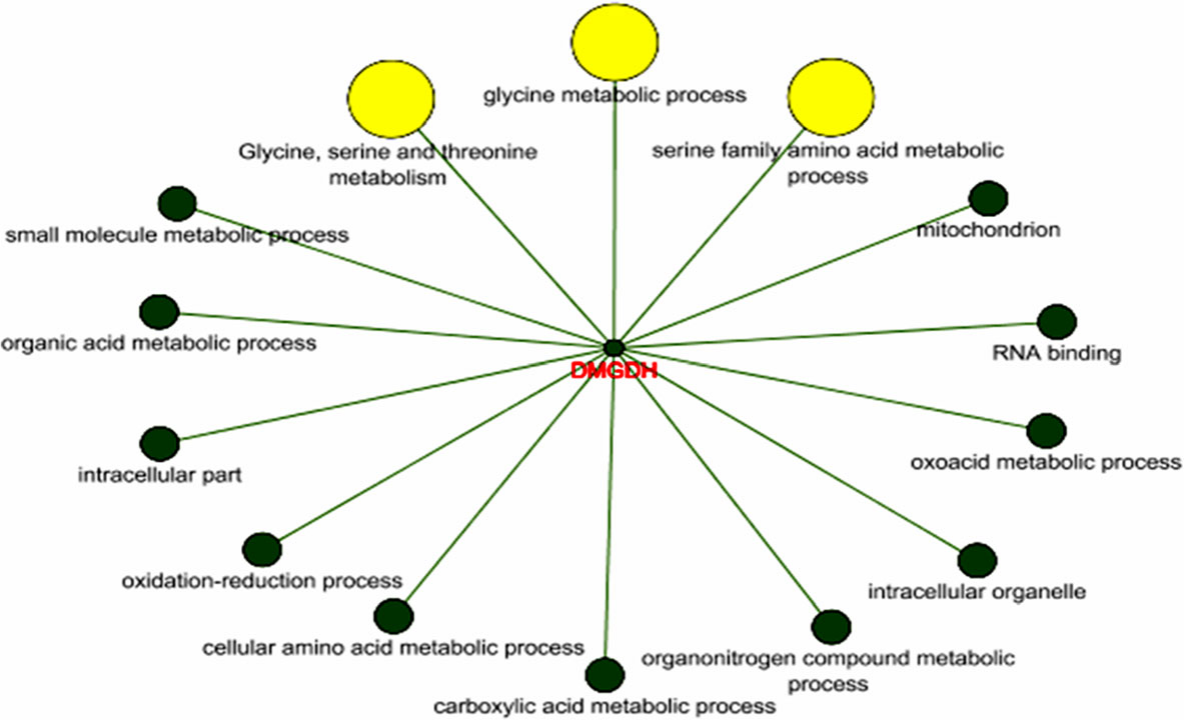
Enrichment analysis of the DMGDH gene with the ClueGO plug-in. The yellow circlels highlight the biological processes and serine and glycine metabolic pathway in which this gene is involved

Using the same programs, the DMGDH gene (dimethylglycine dehydrogenase) was identified as a member of the “glycine, serine and threonine metabolism” pathway (Fig. 2). Glycine makes up about one-third of the helical polypeptide chains of collagen [30]. On the other hand, according to Bailey [2], collagen is degraded by serine proteases, with serine also being part of the glycine metabolic pathway, and by cysteine proteases whose metabolic pathway (“cysteine and methionine metabolism”) is associated with the DMGDH pathway. In the present study, the transcript of this gene was expressed more in tender meat.

**Fig. 3:**
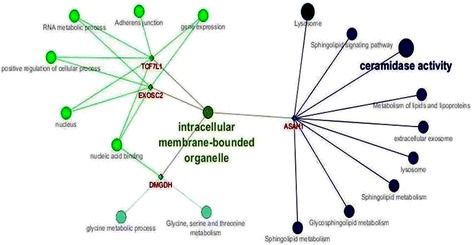
Enrichment analysis of the TCF71, EXOSC2, DMGDH and ASAH1 genes with the ClueGO plug-in

Figure 3 illustrates the interrelationships between the TCF7L1, EXOSC2, DMGDH and ASAH1 transcripts obtained by enrichment analysis. This analysis shows that the main link between these genes is the cell component called “intracellular membrane-bound organelle”. This category refers to structures found inside the cell such as the nucleus and mitochondria [10]. Gene expression analysis in Angus cattle also showed a relationship between meat tenderness and this cell component category [59]. The genes identified in this study are related to actin-myosin assembly, collagen synthesis, lipid accumulation, and serine and glycine metabolic pathways [2, 22, 30, 38].

### Validation of differentially expressed genes

**Fig. 4:**
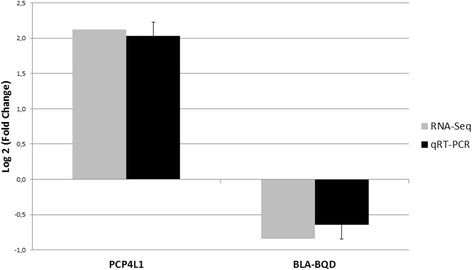
Comparison of the relative expression values of two differentially expressed transcripts obtained by RNA-Seq and qRT-PCR

The relative expression values (log_2_) of the transcripts were similar for the two techniques used, RNA-Seq and qRT-PCR, with values of 2.12 and 2.03 (standard deviation = 0.89) for PCP4L1 and of −0.84 and −0.644 (standard deviation = 0.44) for BoLA-BQD, respectively (Fig. 4). Similar to the RNA-Seq analysis, higher expression of the PCP4L1 and BoLA-BQD genes was observed in the tender and tough meat groups, respectively. Thus, these transcripts showed similar patterns of mRNA abundance in the RNA-Seq and qRT-PCR analyses, with the same direction of expression (i.e., up-regulated and down-regulated, respectively, in relation to the tender meat group).

## Discussion

A higher proportion of Zebu genes in cattle herds considerably reduces meat tenderness when compared to taurine breeds. In Brazil, the herd mostly consists in Zebu cattle, mainly Nellore, then improve meat tenderness is very important, because for the beef export market, in which Brazil plays an important role, tenderness is paramount in determining the value of the product.

Gene expression studies have been used as a tool to identify gene candidates and metabolic pathways related to traits of economic interest. In the present study, the USP32 (ubiquitin specific peptidase 32) transcript was expressed more in tender meat. Members of the ubiquitin-proteasome system are important during the transformation of muscle into meat. These proteins are involved in proteolysis, causing the degradation of myofibrillar proteins in muscle cells [47].

In a genome-wide association study (GWAS) of Nellore cattle using different meat tenderness measures, Tizioto et al. [52] identified genes of the USP family, including USP32. Another study on cattle also associated genes of the USP family with meat tenderness. In Wagiu cattle, the USP2 gene was strongly associated with meat tenderness [12] and gene expression analysis in Nellore cattle showed that the USP2 gene was expressed more in tender meat samples [20].

The functional categories cell junction, regulation of cell communication and intrinsic component of membrane are related to the binding, communication and transport of molecules between cells [10]. Among the transcripts related to these categories, CTNNB1 (catenin - cadherin-associated protein beta 1), which was expressed more in tender meat, is involved in the same metabolic pathway as actin and myosin. Actin and myosin are the proteins found in thin and thick myofilaments, respectively, which form the myofibril that is responsible for muscle contraction. These proteins are the most abundant in the mechanism of muscle contraction, accounting for 52 to 56% of all muscle proteins [48].

Each actin filament binds to the plasma membrane of the cell through a structure, called focal contact. This structure consists of binding proteins and of a transmembrane protein that are products of the “focal adhesion” pathway to which the CTNNB1 and TCF7L1 (transcription factor 7 like 1) genes belong. On the outer side of the cell, in the extracellular matrix, the transmembrane protein binds to a collagen fiber [14, 23]. According to Bailey [2], a direct association exists between collagen content and the toughening of meat. However, in the present study, the CTNNB1 and TCF7L1 transcripts were expressed more in tender meat.

The SYP (synaptophysin) transcript, which was expressed more in tender meat, encodes an integral membrane protein found in small synaptic vesicles. In a study on rats, [44] showed that the phosphorylation of synaptophysin is calcium dependent. The authors observed a four-fold increase in serine phosphorylation of synaptophysin in the presence of the calmodulin-calcium complex. According to Bailey et al. [2], serine proteases are responsible for the degradation of collagen, which, in turn, directly influences meat tenderness. In addition, calcium is essential for muscle contraction by acting as a catalyst of enzymatic proteolytic activity, which is directly related to the process of meat tenderization [37].

The AT2 transcript, which encodes angiotensin II, was expressed more in tender meat. This protein is involved in vasoconstriction and regulates the secretion of aldosterone, which, in turn, stimulates the reabsorption of sodium by the kidneys. In this respect, after slaughter and during bleeding, angiotensin is activated to restore blood pressure. The result of these stimuli is the depolarization of the cell membrane, altering the distribution of sodium and potassium, in addition to permitting the flow of calcium ions [43]. In a study on crossbred cattle (Luxi-Simmental), Zhong-Liang et al. [60] observed a decline in shear force after the injection of angiotensin II into the carcass for 7 days after slaughter. Bongiorni et al. [8], studying gene expression in longissimus dorsi muscle of Italian Maremmana and Chianina breeds, also found the differential expression of genes to be related to sodium and potassium flow.

The functional category “catalytic activity” is related to increases in the velocity of a biochemical reaction at physiological temperatures [10]. Some reactions that occur during the postmortem period depend on calcium and cellular pH, which decrease in the first 24 h after slaughter [25]. A member of this functional category is ASAH1 (N-acylsphingosine amidohydrolase (acid ceramidase) 1), which belongs to a family of hydrolases that catalyze the synthesis and degradation of ceramide into sphingolipid and free fatty acid and are acid pH dependent [32]. A genetic deficiency in ASAH1 that reduces its catalytic activity causes a lysosomal sphingolipid storage disorder characterized by the accumulation of lipids in cells and tissues throughout the organism [38]. ASAH1 also belongs to the “sphingolipid signaling pathway” and “sphingolipid metabolism” categories in which serine is also involved, with serine protease degrading collagen [2]. Thus, ASAH1, which was expressed more in tender meat, may be related to the process of meat tenderization.

Another member of the “catalytic activity” category is HMOX1 (heme oxygenase 1), which was expressed more in tough meat. This gene encodes a protein involved in the metabolism of porphyrins, molecules whose catalytic activity is activated by iron [35]. Porphyrins are precursors of hemes, the main components of hemoglobin, myoglobin and cytochromes which are responsible for the transport of oxygen and electrons in tissues [36].

The C-type lectin (CLEC) family comprises calcium-dependent carbohydrate-binding protein domains that are involved in cell-cell adhesion [15]. In the present study, the CLEC4G and CLEC12A transcripts were expressed more in tender meat. GWAS in Nellore cattle demonstrated an association of the CLEC12A gene with different meat tenderness measures [52].

The IQCG transcript (IQ motif containing G), which was expressed more in tender meat, encodes a protein that functions as a binding site for different proteins, including myosin light chains and calmodulins. Calmodulin phosphorylates myosin, a process that permits the sliding of fibers and muscle contraction. In this case, calcium present in the reaction, binds to calmodulin, attached to IQ motif, and stimulates the ATPase activity of myosin [42]. According to Duston [16], in addition to factors such as collagen content, the structure and state of contraction of myofibrils (which mainly consists of myosin and actin) directly affect meat tenderness.

The protein encoded by the PNP transcript (purine nucleoside phosphorylase), which was expressed more in tough meat, plays a role in nicotinate and nicotinamide metabolism. Nicotinate (niacin or vitamin B3) is a precursor of NAD^+^ and NADP^+^ coenzymes, which are essential for the production of ATP in the cell [28].

Numerous structural changes and biochemical events occur in the first 24 h after slaughter of the animal, which are responsible for the conversion of muscle into meat [25]. In the early postmortem stages, ATP levels are maintained constant by the conversion of ADP plus phosphocreatine into ATP and oxygen supply ceases because of the cessation of blood circulation. At this stage, slow production of lactate is observed and the onset of rigor mortis occurs (slow phase). The decrease in phosphocreatine levels characterizes the rapid phase, which consists of a rapid decline in available ATP that is used as an energy reserve after the consumption of glycogen and other carbohydrates and is therefore hydrolyzed again to ADP. The scarcity of ATP during this phase is accompanied by the release of calcium ions into the myofibrillar space, which causes muscle shortening with a direct influence on meat tenderness [5].

Another event that occurs during this phase is the anaerobic conversion of glycogen into glucose, producing lactate and reducing the pH of the medium. In addition, the transport of sodium and potassium across the cell membrane, which uses the energy released by the hydrolysis of ATP into ADP, is impaired because it occurs against the concentration gradient. The protons generated during the hydrolysis of ATP into ADP cause a significant decline in intracellular pH [3]. According to Darrel et al. [13], this drop in pH directly influences the final tenderness of meat, especially during the process of aging.

According to Koohmaraie et al. [26], calcium is responsible for the activation of calpains and calpastatins (calcium-dependent cysteine proteases) and calpain I has been shown to be the main enzyme responsible for postmortem tenderization of meat by degrading cytoskeletal proteins that confer the structural integrity of the myofibrillar matrix. Nevertheless, in the present study, the calpain and calpastatin genes were not differentially expressed in the tender and tough meat groups. This finding might be explained by the fact that the amount of calpastatin in cells is higher 24 h after slaughter [43] and in this study the samples were collected immediately after cleaning the carcasses. Other GWAS and gene expression studies of muscle tissue in Nellore cattle also found no relationship between meat tenderness and calpain or calpastatin [20, 52].

The EXOSC2 transcript, which encodes exosome component 2, was expressed more in tender meat. According to Jong et al. [22], this gene is related with collagen activity in humans. This found could indicated a relationship between this genes and collagen activity in bovines, because there is a direct association exists between collagen content and the toughening of meat [2].

The ZKSCAN2 transcript (zinc finger with KRAB and SCAN domains 2), which was expressed more in tender meat, is vertebrate specific and synthesizes zinc finger proteins that bind through an N-terminus to the SCAN domain (dimerization motif). The function of this gene is not well known, but zinc finger proteins have been associated with the regulation of growth factor transcription and lipid metabolism [45].

In cattle, the main histocompatibility complex class II is called BoLA-DQB (bovine leukocyte antigen) [24]. In the present study, the BoLA-DQB transcript was expressed more in tough meat. We found no studies investigating the association of this gene with meat tenderness. However, this gene has been associated with growth traits in Holstein and beef cattle (Angus, Charolais, Hereford, Limousin, Simmental); [4, 49] and, according to Koohmaraie et al. [27], animals with higher growth rates have more palatable and more tender meat.

When we compared this study with a GWAS study for meat tenderness using the same Nellore population, we do not found common genes between them, but there were some shared functions related to phosphorylation and catalytic activity [33]. These functions are related with oxygen and calcium transport, and collagen degradation, important processes for the the toughening of meat, especially after slaughter. In a GWAS study using another Nellore cattle population, Tizioto et al. [52] identified regions that influence tenderness at three different time points (24 h and 7 and 14 days after slaughter). Some of the genes reported by these authors were also identified in the present study, such as CLDN19, CLEC12A and USP32. In addition to these genes, the authors reported an association of genes belonging to the family of BoLA-BQD, CTNNB1, EXOSC2 and IQCG transcripts and meat tenderness.

## Conclusions

Global gene expression analysis in animals phenotypically divergent for meat tenderness identified genes related to ubiquitin metabolism, transport of molecules such as calcium and oxygen, acid-base balance, collagen synthesis, actin and myosin, and fat accumulation. These results contribute to the understanding of the molecular mechanisms involved in the meat tenderization process, at the time of slaughter, and to the development of strategies to select animals with more tender meat.

## Methods

### Animals and sample collection

Meat samples were collected from 132 intact male (non castrated) Nellore animals belonging to the same contemporary group (i.e., animals that remained together from birth to slaughter). The animals were from the Capivara Farm that participates in the Qualitas Nelore Breeding Program. All animals were finished in feedlots for, approximately, 90 days and slaughtered at an average age of 731 ± 81 days on the same day and under the same conditions.

The slaughter occurred in a commercial plant, under usual process in Brazilian beef industry: the animals are slaughtered and the half-carcasses are refrigerated by 24 h. After that, the carcass is deboned, frozen and commercialized. All samples were frozen and none of them was aged.

For RNA, muscle tissue (*longissimus dorsi*) samples were collected immediately after slaughtering and stored in 15-mL Falcon tubes containing 5 mL RNA holder (BioAgency, São Paulo, SP, Brazil) at −80 °C until the time for total RNA extraction. Additionally, for shear force measurements, a *longissimus* muscle sample was removed during deboning, after 24 h in a cold chamber, between the 12th and 13th rib of each left half-carcass.

Transcriptome studies show the genes expressed in a specific time for a specific cell, i.e. it shows which genes are been expressed at the moment of the sample collection. So, we have chosen to study the gene expression related with tenderness using the phenotype measured closest to the sample collection for RNA extraction, that is, after 24 h postmortem.

### Analysis of shear force

Longissimus dorsi samples measuring 2.54 cm in thickness were obtained for analysis of tenderness. The standardized procedure proposed by Wheeler et al. [58] was used for shear force determination in a mechanical Salter Warner-Bratzler Shear Force device. The samples analyzed were not submitted to any type of aging process. From this analysis (*n* = 132), 40 samples derived from animals extreme for meat tenderness (20 with tender meat and 20 with tough meat) were selected. The Student t-test implemented in the R environment [41] was applied to verify differences between the tender and tough meat groups (Table 2).

**Table 2:** Number of animals (N), mean, standard error, minimum and maximum of meat tenderness measured by shear force (kgf/cm^2^)

	N	Mean	Standard error	Minimum	Maximum	*P*-value
Tender meat	20	4.36	0.34	3.51	4.80	8.40e-17
Tough meat	20	8.22	1.13	7.33	11.15	

### RNA sequencing

Total RNA was extracted from the samples obtained from the extreme animals selected (*n* = 40). Muscle tissue (longissimus dorsi) samples that were collected immediately after slaughter and stored in 15-mL Falcon tubes containing 5 mL RNA holder (BioAgency, São Paulo, SP, Brazil) at −80 °C were used to extract total RNA. An average of 50 mg of the muscle tissue previously stored in RNA holder (BioAgency, São Paulo, SP, Brazil) was used for extraction with the RNeasy Lipid Tissue Mini Kit (Qiagen, Valencia, CA, USA) according to manufacturer recommendations. The purity of the extracted RNA was determined by reading absorbance in a NanoDrop 1000 spectrophotometer (Thermo Fisher Scientific, Santa Clara, CA, USA, 2007). The quality of the total RNA extracted was evaluated in an Agilent 2100 Bioanalyzer (Agilent, Santa Clara, CA, USA, 2009) and its concentration and contamination with genomic DNA were measured in a Qubit® 2.0 Fluorometer (Invitrogen, Carlsbad, CA, USA, 2010).

Sequencing (RNA-Seq) was performed on an Illumina HiSeq 2500 System. Messenger RNA was obtained from the total RNA extracted and libraries containing 200 bp fragments were constructed and pooled to perform multiplexing sequencing. The reads obtained were paired-end of 2 × 100 bp.

### Sequence processing and alignment

The sequence data generated with the Illumina HiSeq 2500 System were converted into FastQ format, using the Casava software (https://support.illumina.com/downloads/casava_18_changes.html). The computational analyses were performed on CyVerse platform [19].

First, sequenced fragments (reads) of low quality were trimmed using the Sickle program (github.com/najoshi/sickle). The TopHat2 v2.0.9 program [54] was then used to map the fragments and to align them with the bovine reference genome (UMD3.1) available in the NCBI database (http://www.ncbi.nlm.nih.gov/genome/?term=bos+taurus). For each library, a .bam file containing the aligned reads in relation to the reference genome was generated.

### Assembly and quantification of the transcripts

The Cufflinks2 v2.1.1 program [55] was used to assemble the aligned reads of each sample and to estimate the number of transcripts, expressed as fragments per kilobase of transcript per million reads mapped (FPKM). The Cufflinks2 result per sample was concatenated in a single file with the Cuffmerge2 v2.1.1 program, which was used as a reference in the differential gene expression analysis.

### Differential gene expression analysis

Using the Cuffdiff2 v 2.1.1 program [53, 55], the sequence alignment files generated (.bam) were divided into two contrasting groups according to meat tenderness. The FPKM values of each transcript were calculated for each sample. The Cuffdiff2 program uses a *t*-test for the calculation of *p*-values. False discovery rates (FDR) were controlled by the Benjamini-Hochberg procedure considering a q-value of less than 5%.

The CummeRbund package [55], implemented in the R environment [41], was used for exploration and visualization of the data obtained and generate PCA and boxplot graphics.

### Annotation of differentially expressed genes

The Database for Annotation, Visualization, and Integrated Discovery (DAVID) v6.7, which consists of an integrated system of biological databases and analytical tools designed to systematically extract the biological meaning from a large list of genes and/or proteins [21], was used to annotate and interpret the lists of differentially expressed genes. The Functional Annotation Tool was used for this purpose, which determines the most relevant Gene Ontology (GO) terms for each list of genes. The Functional Annotation Clustering algorithm was applied to generate annotations of functional groups. DAVID pathway mapping was used to identify metabolic pathways in which the differentially expressed genes are involved.

The ClueGo plug-in of the Cytoscape program was used to visualize non-redundant biological terms for genes in functionally grouped networks [6].

### Validation of differentially expressed genes

Real-time quantitative PCR (qRT-PCR) was used to validate the differential expression of the genes identified by RNA-Seq analysis. All the 40 RNA samples used in the RNA-Seq analyses was used to validate the data by qRT-PCR. Two differentially expressed genes were chosen randomly for this purpose: bovine leukocyte antigen (BoLA-DQB) and Purkinje cell protein 4-like 1 (PCP4L1). In addition to these genes, three reference genes were chosen and quantified by qRT-PCR, as proposed by Vandesompele et al. [56], to normalize the data. The RNA-Seq technique detected no differences in the expression of the beta-glucuronidase (GUSB), hypoxanthine phosphoribosyltransferase 1 (HPRT1) and TATA box binding protein (TBP) genes between the groups studied and these genes were therefore chosen as housekeeping genes and were tested by qRT-PCR.

The method (conditions and equipment) described by Fonseca et al. [18] was used for validation of the differentially expressed genes by qRT-PCR: One μg total RNA was used to synthetize the first complementary DNA (cDNA) strand using SuperScript III First-Strand Synthesis SuperMix for qRT-PCR (Invitrogen). To design the primers (Table 3), the Primer Express 3.0 software (Applied Biosystems, 2004) was used and the GenBank database (http://www.ncbi.nlm.nih.gov) was accessed to obtain the mRNA nucleotide sequences. The primers specificity was tested by NCBI BLAST algorithm (https://blast.ncbi.nlm.nih.gov/Blast.cgi). Genorm (https://genorm.cmgg.be/) and Expression Suite softwares v1.0 (Applied Biosystems, Foster, CA, USA, 2012) were used to test the expression stability of the housekeeping genes.

**Table 3:** Sequence of the forward (F) and reverse (R) primers used in the qRT-PCR assays

Gene	Locus	Sequence (5′ – 3′)	Tm (° C)^a^	Amplicon size (bp)
BoLA-DQB F	23:25,855,145–25,863,052	CATCACAGGAGCCAGAAG	80	64
BoLA-DQB R		GCAAACACCAATCCCAAAAT		
PCP4L1 F	3:8,249,980–8,276,724	ATCTCCAGCAACCAACCAGG	82	119
PCP4L1 R		TCTCTGTTTCTGGTGCCGTC		
GUSB F	25:28,101,456–28,233,126	CAGGGCGGGATGCTCTA	84	93
GUSB R		GTTGTCGGAGAAGTCGGC		
HPRT1 F	3:103,318,856–103,327,860	TGATGAAGGAGATGGGTGGC	83	81
HPRT1 R		CCAACAGGTCGGCAAAGAAC		
TBP F	9:105,686,118–105,690,814	ATAGTGTGCTGGGGATGCTC	80	114
TBP R		GTGGGAGGCTGTTGTTCTGA		

^a^Denaturation temperature of the amplicon

All qRT-PCR reactions were done with 7500 Real-Time PCR (Applied Biosystems, 2009). For these reactions we used: 0.1 μg cDNA; 1X SYBR Green Master Mix and forward and reverse primers. Primers concentrations were determined by titration: 600 nanoMolar (nM) forward and reverse primers (600/600) for BoLA-BQD and GUSB; 300 nanoMolar (nM) forward and reverse primers (300/300) for HPRT1 and 100 nanoMolar (nM) forward and reverse primers (100/100) for PCP4L1 and TBP. The analyses were performed in triplicate. For each gene (target and housekeeping), we included a negative and a positive control in every reaction.

Serial dilutions of cDNA (1:5) were used to build a standard curve and to calculate the qRT-PCR efficiency for each gene. Only PCR primers showing an efficiency of 90–110% were used [31].

The amplification conditions were: 40 cycles at 50 °C for 2 min, 95 °C for 10 min, and 60 °C for 1 min. Dissociation analyzes were performed to monitor the reactions specificity.

For the housekeeping genes, the geometric means of Ct values were calculated [56]. For the analysis of relative expression, a mixed linear model was fitted [50]:

$$ {\mathrm{Y}}_{\mathrm{gikr}}={\mathrm{T}}_{\mathrm{ig}}+{\mathrm{D}}_{\mathrm{ik}}+{\mathrm{e}}_{\mathrm{gikr}} $$

where: Y_gikr_ is the Ct obtained from the thermocycler software for gene *g*, in the *rth* well of the plate (referring to the technical replicate) in a sample obtained from animal *k* of treatment *i* (low or high meat tenderness group). T_ig_ is the group of animals effect *i* (low or high meat tenderness group) on the expression of gene *g*; D_ik_ is a random sampling specific effect which captures differences between samples shared by genes, particularly those affecting RNA concentration such as different extraction and amplification efficiency, and e_gikr_ is a residual effect.

## Additional files

Additional file 1: Table S1. Samples number (N), classification of the sample, shear force (kgf/cm^2^), number of transcripts aligned in pairs (N reads), and percentage of transcripts aligned in pairs (% reads). (DOCX 16 kb)

Additional file 2: Figure S1. Expression profile of reference genes in the experimental groups (tender and tough meat). (TIFF 176 kb)

Additional file 3: Figure S2. Box plot of expression values (log10 FPKM) obtained for the groups studied (tender and tough meat). (TIFF 162 kb)

Additional file 4: Figure S3. Principal component analysis (PCA) of the transcripts found in the tender (red) and tough (blue) meat groups. (TIFF 215 kb)

Additional file 5: Table S2. Enriched GO terms obtained with the DAVID software for differentially expressed genes. (XLSX 14 kb)

## Abbreviations

ADP: Adenosine Diphosphate
ASAH1: N-Acylsphingosine Amidohydrolase (Acid Ceramidase) 1
AT2: Angiotensin II Receptor Type 2
ATP: Adenosine Triphosphate
BoLA-DQB: Major Histocompatibility Complex, Class II, DQ Beta
cDNA: Complementary DNA
CLDN19: Claudin 19
CLEC: C-Type Lectin Family
CLEC12A: C-Type Lectin Domain Family 12 Member A
CLEC4G: C-Type Lectin Domain Family 4 Member G
Ct: Threshold Cycle
CTNNB1: Catenin - Cadherin-Associated Protein Beta 1
DMGDH: Dimethylglycine Dehydrogenase
EXOSC2: Exosome Component 2
FDR: False Discovery Rates
FPKM: Fragments Per Kilobase Of Transcript Per Million Reads Mapped
GO: Gene Ontology
GUSB: Glucuronidase Beta
GWAS: Genome-Wide Association Study
HMBS: Erythrocyte Hydroxymethylbilane Synthase
HMOX1: Heme Oxygenase 1
HPRT1: Hypoxanthine Phosphoribosyltransferase 1
IQCG: IQ Motif Containing G
NAD^+^: Nicotinamide Adenine Dinucleotide
NADP^+^: Nicotinamide Adenine Dinucleotide Phosphate
nM: nanoMolar
PCA: Principal Component Analysis
PCP4L1: Purkinje Cell Protein 4 Like 1
PGK1: Phosphoglycerate Kinase 1
PNP: Purine Nucleoside Phosphorylase
qRT-PCR: Quantitative Real Time Polymerase Chain Reaction
RNA-Seq: RNA Sequencing
SYP: Synaptophysin
TBP: TATA-Box Binding Protein
TCF7L1: Transcription Factor 7 Like 1
USP: Ubiquitin Specific Peptidase Family
USP2: Ubiquitin Specific Peptidase 2
USP32: Ubiquitin Specific Peptidase 32
ZKSCAN2: Zinc Finger With KRAB And SCAN Domains 2
