# Supplementary material for: Differences in global gene expression in muscle tissue of Nellore cattle with divergent meat tenderness
Source: BMC Genomics. 2017 Dec 4;18:945. doi: 10.1186/s12864-017-4323-0 (PMC5716225; doi:10.1186/s12864-017-4323-0)
Supplement: Supplementary file 1 — Samples number (N), classification of the sample, shear force (kgf/cm2), number of transcripts aligned in pairs (N reads), and percentage of transcripts aligned in pairs (% reads). (DOCX 16 kb) [file 12864_2017_4323_MOESM1_ESM.docx]

**Table 1** Sample number (N), classification of the sample, shear force (kgf/cm^2^), number of transcripts aligned in pairs (N reads), and percentage of transcripts aligned in pairs (%reads).

| **N** | **Sample** | **Shear force** | **N reads** | **% reads** |
| --- | --- | --- | --- | --- |
| 1 | Tender | 3.51 | 29,142,454 | 92.9 |
| 2 | Tender | 3.70 | 16,436,994 | 81.2 |
| 3 | Tender | 3.89 | 27,921,731 | 91.8 |
| 4 | Tender | 4.13 | 13,249,012 | 83.5 |
| 5 | Tender | 4.25 | 26,676,373 | 90.9 |
| 6 | Tender | 4.29 | 27,973,797 | 92.1 |
| 7 | Tender | 4.30 | 31,536,799 | 90.7 |
| 8 | Tender | 4.35 | 26,464,154 | 91.0 |
| 9 | Tender | 4.36 | 23,103,200 | 90.5 |
| 10 | Tender | 4.39 | 27,916,395 | 91.6 |
| 11 | Tender | 4.40 | 21,047,587 | 80.7 |
| 12 | Tender | 4.44 | 23,401,954 | 90.8 |
| 13 | Tender | 4.45 | 25,441,371 | 91.2 |
| 14 | Tender | 4.49 | 28,302,040 | 91.0 |
| 15 | Tender | 4.51 | 24,469,269 | 90.7 |
| 16 | Tender | 4.65 | 11,255,970 | 85.0 |
| 17 | Tender | 4.76 | 12,742,151 | 82.2 |
| 18 | Tender | 4.76 | 31,369,007 | 92.0 |
| 19 | Tender | 4.79 | 26,789,884 | 80.2 |
| 20 | Tender | 4.80 | 43,329,981 | 92.4 |
|  | **Tender mean** | 4.36 | 24,928,506 | 89.0 |
| 21 | Tough | 7.33 | 31,432,600 | 90.9 |
| 22 | Tough | 7.35 | 27,535,877 | 91.9 |
| 23 | Tough | 7.43 | 29,142,454 | 92.9 |
| 24 | Tough | 7.48 | 10,382,695 | 88.2 |
| 25 | Tough | 7.50 | 32,740,303 | 93.0 |
| 26 | Tough | 7.51 | 13,046,954 | 87.7 |
| 27 | Tough | 7.65 | 31,400,341 | 92.0 |
| 28 | Tough | 7.84 | 11,158,756 | 87.3 |
| 29 | Tough | 7.85 | 17,238,643 | 88.2 |
| 30 | Tough | 7.86 | 26,323,144 | 91.2 |
| 31 | Tough | 7.90 | 31,737,792 | 92.0 |
| 32 | Tough | 7.99 | 24,596,119 | 90.9 |
| 33 | Tough | 8.00 | 18,630,195 | 89.6 |
| 34 | Tough | 8.00 | 14,265,931 | 82.5 |
| 35 | Tough | 8.35 | 23,704,428 | 91.0 |
| 36 | Tough | 8.48 | 30,489,482 | 91.5 |
| 37 | Tough | 8.85 | 23,704,428 | 91.0 |
| 38 | Tough | 9.78 | 14,265,931 | 82.5 |
| 39 | Tough | 10.88 | 19,249,626 | 87.1 |
| 40 | Tough | 11.15 | 12,354,720 | 87.1 |
|  | **Tough mean** | 8.26 | 22,170,021 | 89.0 |
|  | **Overall mean** | 6.31 | 23,630,146 | 88.3 |
