# Supplementary figures and images for: Differences in global gene expression in muscle tissue of Nellore cattle with divergent meat tenderness
Source: BMC Genomics. 2017 Dec 4;18:945. doi: 10.1186/s12864-017-4323-0 (PMC5716225; doi:10.1186/s12864-017-4323-0)

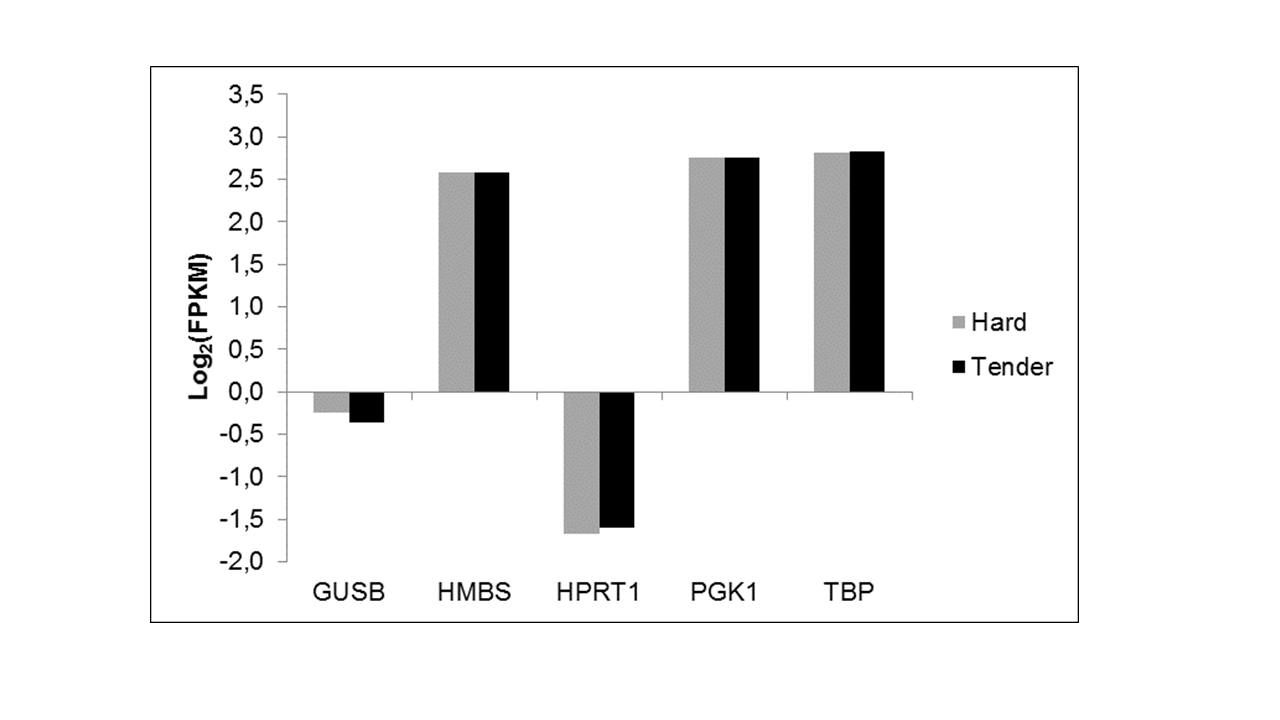

Supplement: Supplementary file 2 — Expression profile of reference genes in the experimental groups (tender and tough meat). (TIFF 176 kb) [file 12864_2017_4323_MOESM2_ESM.tif]

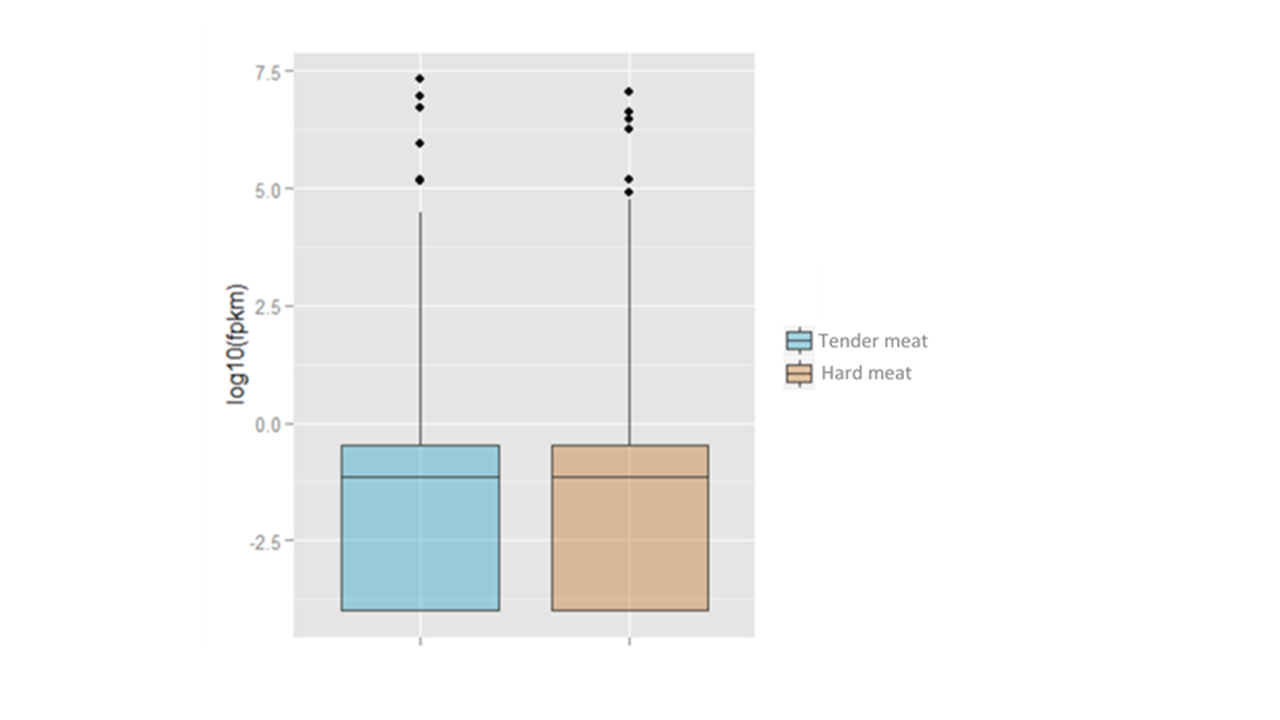

Supplement: Supplementary file 3 — Box plot of expression values (log10 FPKM) obtained for the groups studied (tender and tough meat). (TIFF 162 kb) [file 12864_2017_4323_MOESM3_ESM.tif]

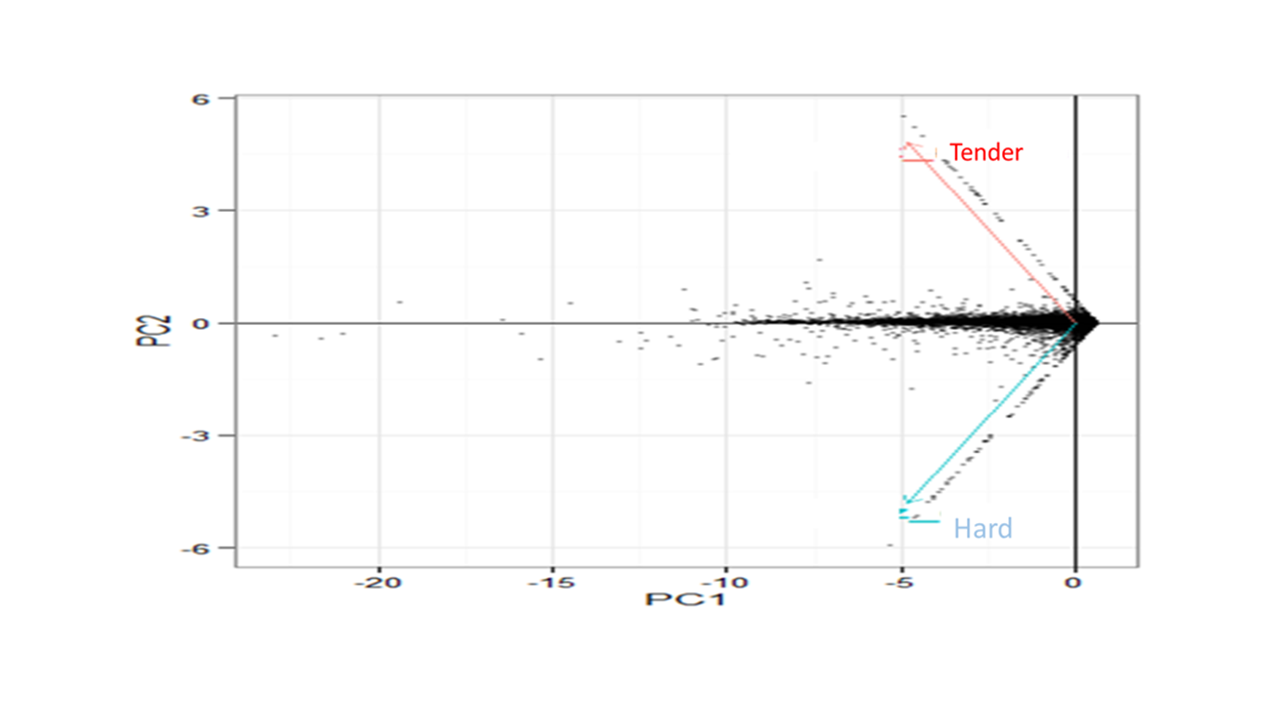

Supplement: Supplementary file 4 — Principal component analysis (PCA) of the transcripts found in the tender (red) and tough (blue) meat groups. (TIFF 215 kb) [file 12864_2017_4323_MOESM4_ESM.tif]
